# Chloroform and Ether

**Published:** 1874-02

**Authors:** J. H. Weir

**Affiliations:** Illinois


### CHLOROFORM AND ETHER.

BY J. H. WEIR, OF ILLINOIS.

I see by the *Boston Medical and Surgical Journal*, Nos. 21 and 22, Vol. LXXXIX, that the medical faculty of that city have had quite an exciting time over two unfortunate deaths, one a boy from inhaling ether, the other a lady, from a mixture of ether and chloroform.

The Boston physicians stepped forward as apoligists for ether, but poor chloroform found no quarters with them.

In both cases from the evidence given, asphyxia was evidently the cause of death, by the unskillful administration of the anaesthetic.

Boston, I believe was the birth place of chloroform, and it is not without *some* honor, save in its native city.

I have used chloroform since it was first placed in the hands of the medical profession. In my first case I was fearful and extremely cautious, using a few drops at a time, and had my patient inhale pure atmospheric air at least half the time, and have never deviated from the same course since. I have administered it to at least one thousand cases of Obstetrics, and more than three times that number in painful and surgical operations from the slightest pain of any part of the body, to convulsions, and from the extracting of teeth to the amputation of a limb, and have never, in at least four thousand cases, have witnessed the slightest injury done by it.

Dr. Cabal, in the Boston case, in answer to a question whether "he thought it dangerous to give ether by inhalation," said, "I don't sir," with, of course, proper precaution," and so *I* say in regard to chloroform.

I have never met with but two cases which seemed to indicate danger, which I detected by the unsteadiness of the eyes and breathing, and I immediately withdiew the chloroform, supplied fresh air, threw a little water in the face, and all was right again, the chloroform was again inhaled in less quantities, and in a short time had them fully insensible to pain, with no unfavorable result. Doctor Curtis says in a communication to the *Boston Medical and*

*Surgical Journal,* page 623, 1873; "Is the peril in any known proportion to the amount of chloroform inhaled?" according to some authors, (Givaldis, page 244) in the majority of fatal cases the dose had been very small, often apparently insignificant, and certainly quite insufficient to produce the benefits of anaesthesia, from fifteen drops to a coffee spoonful.") My opinion is that all fatal cases have inhaled but little, but too rapidly at the start, which produces a nervous shock that interrups respiration after a subject is fully and quietly under its influence, it may be continued for hours without danger, and many ounces may be inhaled in the meantime with impunity.

The leading physicians of Boston seem to think *that* city is the Hub of the Universe, and they are out of patience that the wheel, including Europe, does not revolve at their bidding, abandon the use of chloroform and substitute ether in its stead. I suppose a physician of Boston or vicinity would be indicted for manslaughter, should he suddenly loose a patient, and chloroform should be found in his possession. I never use ether as an anaesthetic, in as much as I think chloroform a much better article, and just as safe, "with of course, proper precaution."

A pint of alcohol, ninety-five per cent., taken at one drink, would kill nine out of every ten adult men or women, but dilute that pint with water, with twenty per cent., and it all can be drunk within twenty-four hours, with perhaps no other injury than thorough intoxication.

Surgeons are in two great haste generally in rendering their patients insensible to pain, better spend thirty minutes to one hour, than run any risk. I almost always spend at least thirty minutes in obtaining the full effect, if my patient exhibits fear or nervous excitement. I give less, and continue longer, giving half the time to the inhalation of pure air, until the excitement passes off.

The boy referred to above, it seems was very much frightened, and had to be held by an assistant, and a cone containing a sponge charged with ether placed over his mouth and nose, compelling the poor struggling victim to breath the ether alone, without a particle of air to oxydise his blood, and absolutely died of suffocation.

The surgeon who would prefer ether to chloroform I suppose would recommend a *dull* knife rather than a sharp one, in the performing of an intricate surgical operation in the vicinity of vita organs. Give me a sharp knife, "with, of course, proper precaution."

One reason that chloroform is more dangerous in incautious hands than ether, is that it is many times stronger, and should be used more cautiously and slower with plenty of fresh air. Another is, that ether is very disagreeable to the patient, and will not be so vigorously inhaled, whereas chloroform is generally very grateful, and is likely to be inhaled too rapidly.

The manner of giving chloroform should be duly considered ; a sponge is not a suitable vehicle, it takes up two much, and lets off too readily, and a cone should never be used, nor anything else, to exclude the atmosphere but a few seconds at a time; the blood must be oxydised, and ether nor chloroform can do it.

I will now give my plan of administering chloroform, which I believe will always give satisfactory results, and without the slightest danger to the patient. I will say I give it to all who desire it, whether they have disease of the heart or lungs, liver, kidneys, nerves or any other organ of the body. I have never found the least difficulty in any of those cases, and if the perfectly strong and healthy, are only to be favored by anaesthetics, and the feeble and diseased to be denied, I see no *mercy* in the thing. I use a clean linen handkerchief or napkin, fold it to three or four inches square, unstop my chloroform vial, place the center of the folded handkerchief in the mouth of the vial, press tightly, turn the vial up once or twice, so as to saturate a point in the center of the fold, (the size of the vial's mouth,) the amount taken up thereby will be about ten drops; then place the face of the first and second fingers of the hand you wish to use, directly over the saturated point of the linen, with the thumb on the opposite side, so as to hold the fold firmly between the fingers and thumb; place the back of the two fingers to the patient's nose, seperate the fingers so as to let the chloroform escape between them, to be inhaled, when you wish to shut it off; close the fingers to prevnnt the escape of the fluid, remove the hand and permit your patient to breathe open air, at equal intervals with the chloroform. The finger being between the linen and the patient's face, prevents the chloroform from burning the skin; thus repeat, turning a little more chloroform on the linen at each time, but should the patient be uneasy and restless, diminish, instead of increasing the quantity, and also the length of time of inhaling it, until quiet is obtained. As the patient becomes quiet, increase the quantity of chloroform. I seldom feel or examine the pulse. I watch the eyes; if they look wild or anxious, desist for a time, if the lids close gently and the balls roll from side to side, all is right, if in the meantime you find your patient quietly getting under its influence, you may hasten it by adding freely of chloroform, and have your patient open, and breath through the mouth, but would not advise inhaling by the mouth at the beginning, nor until the subject be quieting into an anaesthetic state. You can always tell when your patient is fully chloroformed by taking the hand and raising it a little, and letting it drop, if fully under the influence, it will drop as lifeless. This is not only the most economical way of administering chloroform, but is, we think, decidedly the best and saftest, and you have a better knowledge of what quantity your patient is using. Ordinarily one-half ounce of good chloroform properly used, will render a patient insensible to pain.

I believe I can detect any unfavorable effect from the eyes, breathing and countenance, in advance of any change in the pulse, and when the least unfavorable symptoms as anxiety, twitching of muscles or any feature that an ordinary physician can detect as unfavorable to perfect repose, inhalation should then cease for the time. I have worked a full hour to produce the desired point in a nervous and excitable person, yet ordinarily, can reach my end in from ten to fifteen minutes. I look upon chloroform as one of the greatest blessings ever placed in the reach of the medical profession, so as to render *tolerable* relief to human suffering, and I verily believe that any misfortune attending its use is not the fault of chloroform, but of its administration, and just as I would look upon an awkward cut with thc bistory, not that the knife was sharp, but an awkward stroke of the operator. All that is required in my opinion is "*proper precaution.*"

